# 

**Published:** 1956-01

**Authors:** 


### Contents

| | | Page |
| --- | --- | --- |
| DR. ROBERT HUGHES PARRY . . . . | *Editorial* | 1 |
| CONGENITAL BLADDER-NECK OBSTRUCTION: | | |
| PART I . . . . . . . | *Mr. J. P. Mitchell* | 3 |
| PART II . . . . . . . | *Mr. Ashton Miller* | 10 |
| CANCER OF THE UTERUS . . | *Prof. G. Gordon Lennon* | 15 |
| TELLING THE PUBLIC ABOUT MEDICINE . | *Dr. I. Harvey Flack* | 23 |
| CALEB PARRY OF BATH . . . . | *Dr. John Apley* | 30 |
| PRIMARY MYELOSCLEROSIS WITH LEUCO-ERYTHROBLASTIC ANAEMIA . . . . | *Clinical Pathological Conference* | 33 |
| APPOINTMENTS . . . . . . . . | | 37 |

